# Fertility in young patients following treatment for Hodgkin’s lymphoma: a single center survey

**DOI:** 10.1007/s10815-015-0636-6

**Published:** 2015-12-17

**Authors:** Lučka Boltežar, Karlo Pintarić, Barbara Jezeršek Novaković

**Affiliations:** Department of Medical Oncology, Institute of Oncology Ljubljana, Zaloška 2, 1000 Ljubljana, Slovenia; Faculty of Medicine, University of Ljubljana, Vrazov trg 2, 1000 Ljubljana, Slovenia

**Keywords:** Hodgkin lymphoma, ABVD, BEACOPP, Secondary amenorrhea, Fertility following treatment

## Abstract

**Purpose:**

The purpose of this study was to determine the fertility rates following treatment by means of the BEACOPP regimen (regular and escalated) (bleomycin, etoposide, doxorubicin, cyclophosphamide, vincristine, procarbazine, prednisone) as compared to the ABVD regimen (doxorubicin, vinblastine, dacarbazine, bleomycin) in Hodgkin lymphoma patients under the age of 40 at the time of treatment.

**Methods:**

A questionnaire was sent to 180 Hodgkin lymphoma (HL) patients. The questionnaire was composed of questions concerning reproduction and also menopausal and aging symptoms in females and males. The analyses were made using data collected from 123 patients (76 females and 47 males) who returned the questionnaire. All of the patients were treated between 1999 and 2012.

**Results:**

In comparing the ABVD and BEACOPP groups of female patients, the frequency of the therapy-induced amenorrhea and the restored menses following treatment were found to be significantly different statistically (*p* = 0.002 and *p* = 0.012, respectively). The secondary amenorrhea statistically appeared more often in the BEACOPP group (*p* = 0.003) while the cases of achieving pregnancy and having children after chemotherapy were not significantly different (*p* = 0.630, *p* = 0.070, respectively). In comparing the ABVD and BEACOPP treatments in male patients, the only significant difference was in the number of artificially inseminated or in vitro pregnancies achieved in the BEACOPP and escalated BEACOPP group, *p* = 0.008 and *p* = 0.002, respectively. In total, 45.2 % of patients in the ABVD female group, 34.6 % in the BEACOPP female group, 52.6 % in the ABVD male group, and 33.3 % in the male BEACOPP group, respectively, of patients attempting conception post-therapy, had children after chemotherapy.

**Conclusions:**

Based on these high rates of childbirth following BEACOPP chemotherapy, we have concluded that intensified chemotherapy is not a definite predictor of reduced fertility in young HL patients.

## Introduction

Hodgkin lymphoma (HL) is one of the most curable types of lymphoma [1]. Patients are usually young when treated with chemotherapy [2, 3], so being aware of the postliminary consequences of oncologic treatment is crucial. Chemotherapy has been linked to gonadal damage [2–5], with myeloablative therapy increasing the likelihood of childlessness [6]. A study by Meirow et al. on female reproductive organs showed that premature ovarian failure is more frequent in women over 30 years of age, and that this depends on the chemotherapeutic regimen and the particular dose of pelvic irradiation [3]. Especially toxic to the ovarian tissue are alkylating agents [2, 5, 7]. For male patients, Rueffer et al. demonstrated that 70 % of patients suffer from disease-related gonadal dysfunction and semen abnormality even before the initiation of treatment [8]. Today, semen analysis and cryoconservation prior to HL treatment is part of the standard procedure [4, 9].

There have been many studies dealing with fertility in patients following HL treatment performed to the present day [2, 4–6, 10–12]. The dose-escalated BEACOPP (bleomycin, etoposide, doxorubicin, cyclophosphamide, vincristine, procarbazine, and prednisone, where the alkylating agents are cyclophosphamide and procarbazine) regimen has been linked to a higher incidence of secondary amenorrhea than the ABVD regimen (doxorubicin, vinblastine, dacarbazine, and bleomycin, where an alkylating agent is dacarbazine); these two being the most frequently used regimens [3, 4, 13]. The latest largest study by Behringer et al. showed that hormone levels correlated with the intensity of chemotherapy as well as the fact that survivors (following BEACOPP treatment for advanced-stage disease) had the highest risk of symptomatic gonadal dysfunction [13].

The aim of this study was to determine whether the BEACOPP regimen (regular and escalated) compared to the ABVD regimen’s reportedly low fertility rates in Hodgkin lymphoma patients under the age of 40 at the time of treatment.

## Patients and methods

### Patients

The study population included 180 adult patients with HL, who received treatment at the Institute of Oncology in Ljubljana between 1999 and 2012 and were also under age of 40 at the time of treatment. All of these patients were treated with chemotherapy in form of the ABVD (mostly stages I and II) or BEACOPP regimen (mostly stages III or IV until 2011 with 4 cycles of escalated and 4 cycles of regular BEACOPP and, from 2012 on with 6 cycles of escalated BEACOPP). Patients with the nodular lymphocyte predominant type of HL, treated only with irradiation, were excluded from the evaluation as well as patients who received other treatment regimens, i.e., patients treated with pediatric protocols. Patients whose histological diagnosis was inconclusive were also excluded. Informed consent was obtained from all individual participants included in the study and the study was approved by the national ethical committee.

### Questionnaire

We designed a questionnaire including questions about how many pregnancies patients had had before chemotherapy, how many children they had had before chemotherapy, whether or not their menstrual cycle was regular prior to treatment, and whether or not it returned after treatment. Questions concerning the number of children born and pregnancies achieved following treatment were also included, as were the questions regarding embryo/oocyte cryopreservation and in vitro fertilization. For females, the questionnaire, the Menopausal Rating Scale (MRS) [14, 15], was translated into Slovene language comprising the 11 complaints, but without scoring every complaint individually. Patients evaluated their total menopausal symptoms (hot flushes, sweating, unpleasant sensation of impulsive heart palpitations, sleeping disorders, a more depressed mood, irritability, exhaustion, bladder difficulties, vaginal dryness, muscle, and joint pain) with a score from 1 to 10. For males, questions dealing with children engendered before and after chemotherapy were the same. Our male patients were also asked to answer whether or not they had had sperm cryopreservation and whether they had used it for fertilization. The Aging Males’ Symptoms scale (AMS) [16] was also translated into Slovene language scoring the overall complaints on a scale from 1 to 10 (muscle and joint pain, hot flushes, sleeping disorders, excessive need to sleep, a more depressed mood, irritability, exhaustion, loss of physical strength, diminished hair growth, fewer morning erections, loss of sexual desire). The MRS and AMS part of the questionnaire were a subjective measure of patients’ symptoms.

In the questionnaire, questions regarding a partner’s fertility were not asked nor were questions about birth control. The male questionnaire comprised no questions including testosterone treatment of any kind. However, at the end of the questionnaire, all patients were encouraged to comment on any of the questions included as well as state whether they had had as many children as they had wanted as well as whether they were trying to have children post-treatment.

Determination of hormonal levels as well as gynecological examinations to evaluate patients’ fertility status was not a part of this study.

Prior to sending out the questionnaire, each of the 180 patients included in the study population was called in order to obtain her/his consent for the questionnaire. After obtaining consent, the survey was mailed to their address. Four weeks later, the filled in questionnaires (signed and with informed consent) were included in a further analysis, while the non-responders were neither called nor resent the questionnaire. Patients were uncompensated for participation in the study.

### Statistics

The tests used for analyses included the chi-square, unpaired *t* test, Pearson’s correlation test, Mann-Whitney test, and the ANOVA test (for age correlation concerning the number of children born before and after treatment, three subgroups were made: 0—no children, 1—1 child, 2—two or more children). The *p* value <0.05 (two-sided) was considered to be statistically significant. The programs used included the SPSS Statistics (version 19.0, IBM SPSS Inc., USA) and the GraphPad Prism program (version 3.02, GraphPad Software, USA).

## Results

### Patients’ characteristics

A total of 131 (72.78 %) of the 180 patients contacted returned a completed questionnaire. Eighty-two were female (85 % response rate), 49 were male (58 % response rate). Following the aforementioned exclusions, 123 questionnaires were analyzed. Patients were divided into two groups: one that received the ABVD treatment and the other that received the BEACOPP treatment, either regular or escalated. If the patient began with BEACOPP and was later treated with ABVD for whatever reason, he or she was assigned to the BEACOPP group. The BEACOPP group was further subdivided into escalated and normal dosing subgroups for the purpose of statistical analyses. All patients who received at least one cycle of escalated BEACOPP were included into the eBEACOPP subgroup.

There was no significant difference in the disease stage or treatment of those who returned the questionnaire and those who did not (*p* = 0.348 and *p* = 0.244, respectively). Patients answered the questionnaire between 2 and 16 years after treatment with a median of 9 years. The number of children patients had before the treatment had no influence on the selection of therapy regimen. None of the patients were prescribed gonadotropin-releasing hormone analogues during their treatment.

### Female survivors

In the ABVD group, the median age at the time of treatment was 26 years (range 18–39) (Table 1). Two (4.3 %) patients had stage I of the disease, 39 (84.8 %) patients had stage II, one (2.2 %) patient had stage III, and four (8.7 %) patients had stage IV of the disease. Two (4.3 %) patients suffered from constitutional symptoms. Nine patients (19.6 %) had bulky disease and two (4.3 %) had extranodal involvement. Ninety-three point five percent of patients reported a regular menstrual cycle prior treatment. The median number of chemotherapy cycles received was 4 (range 2–8). One (2.2 %) patient had her pelvis irradiated with 23.4 Gy and underwent oocyte cryopreservation prior to treatment. Six patients (13.0 %) relapsed after first line treatment, five received salvage chemotherapy, and one underwent surgical removal of the involved lymph node. All but one, are at present in complete remission (CR).

**Table 1:** Female patients’ characteristics

	ABVD group	BEACOPP group	*p* value
Number of patients	46	30 (19e/11b)	NA
Median age in years at diagnosis (range)	26 (range 18–39)	26.5 (range 20–36)	*p* = 0.869
Stage of disease prior to treatment			
I	2 (4.3 %)	0 (0 %)	NA
II	39 (84.8 %)	10 (33.3 %)	NA
III	1 (2.2 %)	5 (16.7 %)	NA
IV	4 (8.7 %)	15 (50.0 %)	NA
A/B	44 (95.7 %)/2 (4.3 %)	21 (70.0 %)/9 (30.0 %)	*p* = *0.005*
X	9 (19.6 %)	9 (30.0 %)	*p* = 0.408
E	2 (4.3 %)	7 (23.3 %)	*p* = *0.025*
Median number of cycles (range)	4 (range 2–8)	8 (range 4–8)	NA
CR/PR after chemotherapy	8 (17.4 %)/38 (82.6 %)	12 (40.0 %)/18 (60.0 %)	*p* = *0.036*
Irradiation of the pelvis	1 (2.2 %)	0 (0 %)	*p* = 1.000
Number of patients receiving at least 1 cycle of eBEACOPP	0 (0 %)	19 (63.3 %)	NA
Number of patients with children before treatment	17 (37.0 %)	16 (53.3 %)	*p* = 0.154
Regular cycle before treatment	43 (93.5 %)	28 (93.3 %)	*p* = 1.000
Embryo/oocyte cryopreservation	2 (4.3 %)	0 (0 %)	*p* = 0.516
Therapy-induced amenorrhea	17 (37.0 %)	23 (76.7 %)	*p* = *0.002*
Restored menses after treatment	14 (82.4 %)	10 (43.5 %)	*p* = *0.012*
Secondary amenorrhea^b^	3 (6.5 %)	13 (43.3 %)	*p* = *0.003*
All pregnancies after treatment/in vitro fertilization^a^	21/1 (4.8 %)	10/0 (0 %)	*p* = 0.630
Number of patients attempting conception post-treatment	42 (91.3 %)	26 (86.7 %)	*p* = 0.879
Number of patients having children after treatment^c^	19 (45.2 %)	9 (34.6 %)	*p* = 0.573
Menopausal problems/mean score	17 (37 %)/4.65	17 (56.7 %)/6.05	*p* = 0.095

Italic values indicate statistical significance

*A* without constitutional symptoms, *B* with constitutional symptoms, *X* bulky disease, *E* extranodal involvement, *CR* complete remission, *PR* partial remission, *NA* not applicable, *e* escalated, *b* basal

^a^Number of in vitro fertilized pregnancies among all pregnancies after chemotherapy

^b^Percentage of all patients in ABVD and BEACOPP groups

^c^Percentage of patients attempting conception post-treatment

In the BEACOPP group, the median age at the time of treatment was 26.5 years (range 20–36). Ten (33.3 %) patients had stage II of the disease, five (16.7 %) patients had stage III, and 15 (50.0 %) patients had stage IV of the disease. Nine (30.0 %) had constitutional symptoms and nine (30.0 %) had bulky disease. Seven (23.3 %) had an extranodal localization. Ninety-three point three percent of patients reported a regular menstrual cycle prior to treatment. The median number of chemotherapy cycles received was 8 (range 4–8). There was only one relapse (3.3 %) in this group (the patient in question was treated with salvage chemotherapy and is now in remission). The rest of the group achieved CR after first line treatment with chemotherapy or chemoradiotherapy. None of the patients received irradiation of the pelvis.

A higher age in female patients correlated significantly with a higher number of children born before and a lower number of children born after treatment (*p* < 0.001, *p* = 0.032, respectively) (Table 2). There was no difference in age at the time they answered the questionnaire (*p* = 0.905). Regardless of the regimen used, a higher age at the time of treatment also correlated positively with therapy-induced amenorrhea as well as with the presence of menopausal symptoms and correlated negatively with pregnancies achieved after treatment as well as in the birth of children after therapy (*p* = 0.016, *p* < 0.001, *p* = 0.014, *p* = 0.010, respectively). A higher age at the time of treatment in the ABVD group correlated only with a higher number of children prior to the treatment (*p* = 0.002) and also with the presence of menopausal symptoms (*p* = 0.013). However, in the BEACOPP group, a higher age at the time of treatment significantly correlated with a higher number of children born before treatment (*p* < 0.001). A positive correlation was found with therapy-induced amenorrhea and with the presence of menopausal symptoms while a negative correlation was found with both the restored menses and with the birth of children after chemotherapy (*p* = 0.029, *p* = 0.015, *p* = 0.010, and *p* = 0.040, respectively). It did not correlate with the number of achieved pregnancies (*p* = 0.051) or with the severity of menopausal symptoms (*p* = 0.511). None of our patients had been fertilized with a donor egg cell, though one female was in the process of in vitro fertilization with a donor egg at the time of the questionnaire. Only one patient from the ABVD and one from the BEACOPP group reported difficulties in becoming pregnant before chemotherapy. They both bore one child following chemotherapy with one of them (ABVD) selecting the in vitro fertilization procedure.

**Table 2:** Correlations and statistical significance among female patients

	Age at treatment	Age at treatment (ABVD group)	Age at treatment (BEACOPP group)	ABVD vs. BEACOPP	ABVD vs. eBEACOPP	bBEACOPP vs. eBEACOPP
Number of children before treatment	*p* < *0.001* ^a^	*p* = *0.002* ^a^	*p* < *0.001* ^a^	*p* = 0.297^b^	*p* = *0.027* ^b^	*p* = 0.168^b^
Therapy-induced amenorrhea	*p* = *0.016* ^c^	*p* = 0.118^c^	*p* = *0.029* ^c^	*p* = *0.002* ^d^	*p* < *0.001* ^d^	*p* = 0.712^d^
Restored menses after treatment	*p* = 0.062^c^	*p* = 0.348^c^	*p* = *0.010* ^c^	*p* = *0.012* ^d^	*p* = *0.032* ^d^	*p* = 1.000^d^
Secondary amenorrhea	*p* = 0.062^c^	*p* = 0.348^c^	*p* = *0.010* ^c^	*p* = *0.003* ^d^	*p* = 0.494^d^	*p* = 1.000^d^
Pregnancies after therapy	*p* = *0.014* ^c^	*p* = 0.095^c^	*p* = 0.051^c^	*p* = 0.630^d^	*p* = 0.174^d^	*p* = 0.425^d^
Children after therapy (yes/no)	*p* = *0.010* ^c^	*p* = 0.079^c^	*p* = *0.040* ^c^	*p* = 0.070^d^	*p* = 0.155^d^	*p* = 0.225^d^
Number of children after therapy	*p* = *0.032* ^a^	*p* = 0.191^a^	*p* = 0.124^a^	*p* = 0.297^b^	*p* = 0.122^b^	*p* = 0.205^b^
Menopausal symptoms	*p* < *0.001* ^c^	*p* = *0.013* ^c^	*p* = *0.015* ^c^	*p* = 0.095^d^	*p* = 0.984^d^	*p* = 0.454^d^
Severity of symptoms	*p* = 0.665^e^	*p* = 0.983^e^	*p* = 0.511^e^	*p* = 0.657^b^	*p* = 0.766^b^	*p* = 0.836^b^

Italic values indicate statistical significance

^a^ANOVA test

^b^Mann-Whitney test

^c^ *t* test

^d^Chi-square

^e^Pearson’s correlation

The severity of problems in achieving pregnancy before treatment did not differ among ABVD and BEACOPP groups nor did the embryo/oocyte cryopreservation (*p* = 1.000, data not shown and *p* = 0.516 (Table 1), respectively). Only four women in each treatment group reported that they had not tried to conceive a child after the treatment. The therapy-induced amenorrhea was significantly different statistically among the groups and so was the restored menses after treatment (*p* = 0.002 and *p* = 0.012, respectively, Tables 1 and 2). The value of the therapy-induced amenorrhea was only determined for women who had previously had regular cycles. There was no difference in amenorrhea and restored menses among women below and over 30 years of age in joined group analyses (*p* = 0.388). Statistically, secondary amenorrhea after treatment appeared significantly more often in the BEACOPP group (*p* = 0.003, Table 1 and 2) and was also more frequent in patients over 30 years of age (*p* = 0.008). It was defined as an absent (and not only irregular) cycle after treatment. Achieving pregnancy (conception) and having children after chemotherapy were non-significant variables between the two groups (*p* = 0.630, *p* = 0.070, respectively). Only one patient in the ABVD group reported a miscarriage, but had another child after treatment while others birthed live children. Also, no difference was found among the groups in regard to the presence of menopausal symptoms (*p* = 0.095) or severity of these symptoms (*p* = 0.657). In the BEACOPP subgroups, there was no significant difference in any of the aforementioned parameters (Table 2). When comparing the ABVD group and the escalated BEACOPP group, a statistically significant difference was discovered in the number of children born before treatment began (*p* = 0.027) (higher in the eBEACOPP group), in therapy-induced amenorrhea (*p* < 0.001) (more frequent in the eBEACOPP group), and so was the incidence of restored menses after treatment (*p* = 0.032) (less often in the eBEACOPP group). Other parameters were considered insignificant.

### Male survivors

In the ABVD group, the median age at the time of treatment was 28 (range 20–39) (Table 3). Four (14.8 %) patients had stage I and 23 (85.2 %) patients had stage II of the disease. Six (22.2 %) patients had bulky disease and 3 (11.1 %) suffered from constitutional symptoms. One (3.7 %) had extranodal involvement. The median number of chemotherapy cycles was 4 (range 1–6). Only three (11.1 %) patients had CR after first line chemotherapy, while 24 (88.9 %) patients needed additional radiotherapy. One (3.7 %) patient received irradiation of the scrotum with 24 Gy due to the involvement of regional lymph nodes while sperm cryoconservation was not done due to his age (38 years). Three (11.1 %) patients relapsed, two of them received salvage chemotherapy and are now in remission, the third declined chemotherapy and is still under observation without any further treatment. Thirteen (48.1 %) patients reported male aging symptoms. Six out of eight patients who declined sperm cryopreservation had already had children previously and were older than 35 years of age. Only in one patient, the usage of cryopreserved sperm was reported unsuccessful in terms of conception, other six utilizations were successful.

**Table 3:** Male patients’ characteristics

	ABVD group	BEACOPP group	*p* value
Number of patients	27	20 (16e, 4b)	NA
Median age in years at diagnosis (range)	28 (range 20–39)	25.5 (range 16–38)	*p* = 0.054
Stage of disease prior to treatment			
I	4 (14.8 %)	2^a^ (10.0 %)	NA
II	23 (85.2 %)	1^a^ (5.0 %)	NA
III	0 (0 %)	7 (35.0 %)	NA
IV	0 (0 %)	10 (50.0 %)	NA
A/B	24 (88.9 %)/3 (11.1 %)	11 (55.0 %)/9 (45.0 %)	*p* = *0.016*
X	6 (22.2 %)	9 (45.0 %)	*p* = 0.122
E	1 (3.7 %)	2 (10.0 %)	*p* = 0.563
Median number of cycles (range)	4 (1–6)	8 (4–8)	NA
CR/PR after chemotherapy	3 (11.1 %)/24 (88.9 %)	11 (55.0 %)/9 (45.0 %)	*p* = *0.003*
Irradiation of the scrotum	1 (3.7 %)	0 (0 %)	*p* = 1.000
Number of patients receiving at least 1 cycle of eBEACOPP	0 (0 %)	16 (80.0 %)	NA
Number of patients with children before treatment	10 (37.0 %)	8 (40.0 %)	*p* = 1.000
Sperm cryoconservation prior treatment	19 (70.4 %)	18 (90.0 %)	*p* = 0.154
Number of patients achieving pregnancy after therapy/in vitro fertilization^b^	10 (37.0 %)/1 (10.0 %)	6 (30.0 %)/5 (83.3 %)	*p* = 0.753/*p* = *0.008*
Number of patients attempting conception post-treatment	19 (70.4 %)	18 (90.0 %)	*p* = 0.154
Number of patients having children after therapy^c^	10 (52.6 %)	6 (33.3 %)	*p* = 0.554
Aging male symptoms/mean score	13 (48.1 %)/4.60	10 (50.0 %)/3.80	*p* = 1.000

Italic values indicate statistical significance

*A* without constitutional symptoms, *B* with constitutional symptoms, *X* bulky disease, *E* extranodal involvement, *CR* complete remission, *PR* partial remission, *NA* not applicable, *e* escalated, *b* basal

^a^All patients with stage I and II in BEACOPP group were treated with regular-dose BEACOPP

^b^Number of artificially inseminated or in vitro fertilized pregnancies among achieved pregnancies after treatment

^c^Percentage of patients attempting conception post-treatment

In the BEACOPP group, the median age at the time of treatment was 25.5 (range 16–38). Two (10.0 %) patients had stage I of the disease, one (5.0 %) patient had stage II, seven (35.0 %) patients had stage III, and ten (50.0 %) patients had stage IV of the disease. Nine (45.0 %) patients suffered from constitutional symptoms, nine (45.0 %) had bulky disease, and two (10.0 %) had extranodal involvement. The median number of chemotherapy cycles was 8 (range 4–8). None of the patients were irradiated in the scrotal area. Eleven (55.0 %) patients achieved CR after chemotherapy, nine (45.0 %) needed radiotherapy. One (5.0 %) patient relapsed and received salvage treatment and is now in CR. Ten (50 %) patients reported aging male symptoms.

None of the male participants specifically stated that they did not try to have children after chemotherapy with the exception of the ten (eight in the ABVD and two in the BEACOPP group) patients who refused the sperm cryopreservation. A higher age correlated negatively with sperm cryoconservation (Table 4) and with achieving pregnancy following chemotherapy and correlated positively with the number of children born before chemotherapy (*p* < 0.001, *p* < 0.001, *p* = 0.010, respectively). A higher age, however, did not correlate with the presence of aging male symptoms (*p* = 0.382), the severity of these problems (*p* = 0.200), with a higher number of in vitro fertilization procedures necessary to achieve pregnancy (*p* = 0.826) or with a lower number of children born following chemotherapy (*p* = 0.921). The BEACOPP group was significantly younger at the time they answered the questionnaire (*p* = 0.023, data not shown) but not at the time of diagnosis of HL (*p* = 0.054). In the ABVD group, a higher age correlated positively with the number of children born before chemotherapy (*p* < 0.001) and the presence of aging male symptoms (*p* = 0.028), while it correlated negatively with sperm cryoconservation (*p* < 0.001) and with achieving pregnancy after therapy (*p* = 0.043). However, it did not correlate with the severity of aging male symptoms (*p* = 0.806) or the number of children born after therapy (*p* = 0.129). In the BEACOPP group, a higher age correlated only with a higher number of children before treatment (*p* = 0.034) and none of the other parameters.

**Table 4:** Correlations and statistical significance among male patients

	Age at treatment	Age at treatment (ABVD group)	Age at treatment (BEACOPP group)	ABVD vs. BEACOPP	ABVD vs. eBEACOPP	BEACOPP vs. eBEACOPP
Number of children before treatment	*p* = *0.010* ^a^	*p* < *0.001* ^a^	*p* = *0.034* ^a^	*p* = 0.594^b^	*p* = 0.782^b^	*p* = 0.475^b^
Sperm cryopreservation	*p* < *0.001* ^c^	*p* < *0.001*	/	*p* = 0.154^d^	*p* = 0.276^d^	*p* = 1.000^d^
Achieving pregnancy after treatment	*p* < *0.001* ^c^	*p* = *0.043* ^c^	*p* = 0.185^c^	*p* = 0.753^d^	*p* = 0.512^d^	*p* = 0.549^d^
In vitro fertilization/artificial insemination	*p* = 0.826^c^	/	/	*p* = *0.008* ^d^	*p* = *0.002* ^d^	*p* = 0.333^d^
Number of children after therapy	*p* = 0.921^a^	p = 0.129^a^	/	*p* = 0.674^b^	*p* = 0.446^b^	*p* = 0.384^b^
Aging male symptoms	*p* = 0.382^c^	*p* = *0.028* ^c^	*p* = 0.386^c^	*p* = 1.000^d^	*p* = 1.000^d^	*p* = 1.410^d^
Severity of aging male symptoms	*p* = 0.200^e^	*p* = 0.806^e^	*p* = 0.126^e^	*p* = 0.426^b^	*p* = 0.432^b^	*p* = 0.937^b^

Italic values indicate statistical significance

*/* statistical analyses could not be performed due to a small number of subjects in analyzed subgroup

^a^ANOVA test

^b^Mann-Whitney test

^c^ *t* test

^d^Chi-square

^e^Pearson’s correlation

There was no difference between the ABVD group and the BEACOPP group in case of sperm cryopreservation, achieving pregnancy post-treatment, the number of children after therapy or aging male symptoms and its severity (*p* = 0.154, *p* = 0.753, *p* = 0.674, *p* = 1.000 and *p* = 0.426, respectively). The only statistical significance discovered was in the frequency of cryopreserved sperm use for artificial insemination or in vitro fertilization for the achieved pregnancies when comparing the ABVD and BEACOPP group as well as the ABVD and the escalated BEACOPP group (*p* = 0.008, *p* = 0.002, respectively). The cryopreserved sperm was more frequently used for fertilization in the BEACOPP group with 83.3 % of patients requiring it. No other aforementioned parameters were significantly different between the two groups. No statistically significant difference was found in comparing the regular BEACOPP and the escalated BEACOPP groups.

## Discussion

### Female survivors

The patients’ age at the time of treatment correlated with the number of children the women had had before therapy in the joined group, as well as in both divided groups, which is representative of the reproductive age of the female population. Those aged between 25–29 and 30–35 years had the highest birth rates in Slovenia in 2013 [17]. Regardless of the regimen used, a higher age resulted in more therapy-induced amenorrhea and with the presence of menopausal symptoms. A higher age correlated negatively with pregnancy achieved after treatment, children born after therapy, and the number of children born after chemotherapy. In the BEACOPP group, a higher age positively correlated with therapy-induced amenorrhea and with definite secondary amenorrhea and correlated negatively with restored menses and with the number of children born after chemotherapy. With no difference in median age at the time of diagnosis between the two groups, we attributed this difference to the depleted number of follicles in older female patients and therefore increased susceptibility to the toxicities of chemotherapy [11]. For the same reason, we presume that age strongly correlated with menopausal symptoms. The BEACOPP regimen has often been linked to secondary amenorrhea [3, 4, 13] especially due to the alkylating agents used [4, 5] unlike ABVD [12, 13]. Behringer et al. strongly correlated the risk of amenorrhea in BEACOPP patients 4 years after chemotherapy to 30 years of age or above [13]. Our study showed the same and the secondary amenorrhea in the BEACOPP group was more frequent in those above 30 years of age, but this was not so in the ABVD group (*p* = 0.008, *p* = 0.537, respectively, data not shown). In their study, Behringer et al. also correlated 30-year-old BEACOPP patients with the severity of menopausal problems, which has not been proven in our study. Namely, in our study, the *p* value in the BEACOPP group was 0.724 and in the escalated BEACOPP group it was 0.442 (data not shown).

Statistically, the therapy-induced amenorrhea was significantly different among the ABVD and BEACOPP groups (more frequent in the BEACOPP group) as was the restored menses following treatment (more frequent in the ABVD group). Forty-three percent of women in the BEACOPP group and 6.5 % in the ABVD group reported secondary amenorrhea, which is in accordance with the literature [13]. In the German Hodgkin Lymphoma Study Group, a study of secondary amenorrhea was reported in 51.4 % patients who received 8 cycle dosages for escalated BEACOPP [4], whereas in van der Kaaij’s premature ovarian failure study, 34 % of 202 women treated with alkylating chemotherapy and only 3 % of the 151 patients treated with nonalkylating chemotherapy developed premature ovarian failure [2]. Statistically, secondary amenorrhea was significantly different between the ABVD and BEACOPP groups, but not in the case of comparing ABVD to the escalated BEACOPP. This is fascinating as the escalated BEACOPP was expected to do more secondary gonadal damage than other regimens [4, 7, 18]. However, caution is required in such an interpretation as a rather small number of women were included in the basal BEACOPP and escalated BEACOPP group. Moreover, as in the questionnaire, we did not specifically ask whether women were taking oral contraceptives; this fact should also be considered while evaluating amenorrhea.

We found no difference in the pregnancy rate among the groups: 34.6 % of women in the BEACOPP group attempting conception post-treatment reported having children after therapy along with 45.2 % in the ABVD group, respectively, which is almost twice the number reported in Behringer’s study [13]. However, these numbers might have been even higher as a number of patients reported that they have not even tried to have children and therefore they cannot know whether they are infertile. Therefore, our results may perhaps be an underestimation of the actual fertility rate as our study only included a questionnaire without determining hormone levels, consequently, leaving the reduced ovarian reserve unmeasured. Furthermore, the extent of ovarian damage due to salvage therapy has not been separately evaluated in this study, hypothetically contributing additionally to lower observed pregnancy rates. Still, we feel obliged to report a successful conception and a birth of a healthy child following high-dose chemotherapy and autologous stem-cell transplantation in ABVD treated woman. Nevertheless, with no difference in the pregnancy rate following chemotherapy and in children born following therapy, we can conclude that the BEACOPP regimen is not as toxic as previously reported [4, 7, 13], which is also consistent with the Cochrane review of 2011 reporting no differences in fertility following treatment between the ABVD and escalated BEACOPP regimen [19]. Still, a concerning observation was that no women in the BEACOPP group underwent embryo/oocyte cryopreservation and only two (4.3 %) patients in the ABVD group did, which implies that each physician should be more aware of discussing the fertility preservation procedures with his patient prior to treatment. In the last 2 years, almost every young patient under the age of 40 is offered a cryopreservation procedure prior to treatment.

### Male survivors

In male patients, a higher age at the time of treatment correlated with the number of children born before chemotherapy in both regimens analyzed together and separately. Regardless of the regimen used, a higher age at the time of treatment also correlated with a less frequent sperm cryoconservation and a smaller probability of achieving pregnancy after chemotherapy in general. However, it is interesting that a higher age did not correlate with the presence of aging male symptoms or with the severity of these symptoms. The ABVD group was the only group where a higher age correlated with more frequent aging male symptoms with the median age at the time of treatment being 2.5 years more than in the BEACOPP group. Miwa et al. did not find a correlation between age and the aging male symptoms score; however, their study was conducted in ambulatory men and not on cancer patients undergoing treatment [20]. The presence and severity of aging male symptoms did not differ among group analyses, which is consistent with the Behringer’s study [13].

Hormone levels were not measured in our study along with the presence of pretreatment dysspermia as in other studies [13, 21]. Also, questions about steroid therapy after treatment were left out of the questionnaire. However, 52.6 % of men in the ABVD group attempting conception post-treatment and 33.3 % of men in the BEACOPP group reported having children after chemotherapy, 10 % with cryoconservated sperm in the ABVD group, and 83.3 % with cryoconservated sperm in the BEACOPP group, which could be attributed to the higher toxicity of the BEACOPP treatment. In Behringer’s study, only 12 % of patients treated with the BEACOPP regimen reported parenthood after 4 years; 10 % used their cryopreserved sperm [13]. Also, the number of sperm cryoconservation procedures was higher in our study with 70.4 % in ABVD and 90 % in BEACOPP group vs. 30 and 37 %, respectively, in Behringer’s study which could explain patients having more children after therapy in our study [13].

The only statistically significant parameter found was the frequency of cryoconserved sperm use for in vitro fertilization procedures between the ABVD group and the BEACOPP as well as in the ABVD and the escalated BEACOPP regimen. However, as previously mentioned, Rueffer demonstrated that 70 % of patients already suffered from semen abnormality before the initiation of anticancer treatment [8]. Also, the German Hodgkin Study Group reported 89 % of patients with azoospermia and 11 % with other forms of dysspermia after BEACOPP [21]. Since our study did not include the semen analyses and its evaluation before treatment, we cannot conclude whether or not the dysspermia (before or after treatment) was the cause for the statistical difference. Still, the significance in the usage of cryopreservated sperm (more often in the BEACOPP group) could be due to the toxicity of the BEACOPP regimen. However, the number of children born after chemotherapy did not differ between the two groups suggesting that our male patients were likely affected by the gonadotoxic BEACOPP, but were still able to achieve similar rates of parenthood as in the ABVD group by means of assisted reproduction. The stage of the disease prior to treatment, however, was significantly higher in the escalated BEACOPP group, which was the foundation for the selection of more aggressive treatments, but it could also mean that the disease was more systemic, affecting the spermatogenesis itself. Van der Kaaji et al. showed that in early stage HL (stages I and II), 41 % of patients have good quality sperm, 49 % intermediate, and 7 % of patients have poor quality sperm (according to the World Health Organisation classification) [22]. However, their patients did not receive the BEACOPP treatment. The outcome of the chemotherapy, on the other hand, was significantly better in the BEACOPP group than in the ABVD group, indicating a higher level of effectiveness in the BEACOPP regimen and implying the frequent dilemma of weighing a more effective chemotherapy option against reduced fertility afterwards. The other possible cause for a higher frequency of assisted reproduction procedures in the BEACOPP group could also be found in the men’s partners. In the questionnaire, it was not specifically noted whether the in vitro fertilization procedure was performed due to lower male reproductive capacities or female reproductive capacities.

## Conclusion

Although the expected frequency of secondary amenorrhea is high, we can also report a high percentage of pregnancies following treatment with the ABVD and BEACOPP regimens: 45.2 % in the ABVD group and 34.6 % in the BEACOPP group, respectively, of patients attempting conception post-therapy, in comparison with previous reports. High post-therapy fatherhood rates were also observed among male patients with 52.6 % of patients in the ABVD group of patients attempting conception post-therapy and 33.3 % patients in the BEACOPP group, respectively, producing children with a significantly more frequent usage of cryopreserved sperm in the BEACOPP group. Based on our findings, we can conclude that even intensified chemotherapy is not a definite predictor of infertility in young HL patients.

## Abbreviations

A: without constitutional symptoms
ABVD regimen: doxorubicin, vinblastine, dacarbazine, and bleomycin
AMS: Aging Males’ Symptoms scale
ANOVA: analysis of variance test
B: with constitutional symptoms
bBEACOPP: basal BEACOPP
BEACOPP regimen: bleomycin, etoposide, doxorubicin, cyclophosphamide, vincristine, procarbazine, and prednisone
CR: complete remission
eBEACOPP: escalated BEACOPP
E: extranodal involvement
Gy: gray
HL: Hodgkin lymphoma
MRS: Menopause Rating Scale
NA: not applicable
PR: partial remission
SPSS: Statistical Package for Social Sciences
X: bulky disease
